# Heat of Decomposition of Potassium Perchlorate

**DOI:** 10.6028/jres.065A.005

**Published:** 1961-02-01

**Authors:** Walter H. Johnson, Alexis A. Gilliland

## Abstract

The heat of decomposition of potassium perchlorate into potassium chloride and oxygen has been determined in a bomb calorimeter. The process may be represented by the equation:

$$\begin{array}{l} {\text{KClO}}_{4}\left(c\right)\;=\text{KCl}\left(c\right)+2O_{2}\left(g\right),\\ \Delta H{}^{\circ}\left(25{}^{\circ}C\right)=-4.02\pm 0.34\;\text{kj}/\text{mole},\\ \;=-0.96\pm 0.08\;\text{kcal}/\text{mole}. \end{array}$$

Combination of this datum with the heat of formation of KCl(c) gives −103.22 ±0.15 kcal/mole for the standard heat of formation of KClO_4_(c) at 25 °C.

## 1. Introduction

The thermodynamic properties of alkali and light- metal perchlorates have become increasingly important during recent years because of their possible use for sources of oxygen in solid-fuel systems. Potassium perchlorate, for example, contains approximately the same quantity of oxygen per unit volume as liquid oxygen at its boiling point. The release of this relatively large amount of oxygen may be effected by heating to about 550 °C. Some of the other perchlorates decompose at considerably lower temperatures.

An accurate value for the heat of decomposition of KClO_4_ leads to a reliable value for the heat of formation, which serves as a convenient basis for evaluation of the heats of formation of other perchlorates. Potassium perchlorate was chosen because of its stability, ease of purification, and nonhygroscopic character.

## 2. Materials

The potassium perchlorate was reagent-grade material, which was recrystallized twice from water and dried at 135 °C; it was stored in a desiccator over anhydrous magnesium perchlorate. The lot analysis of the material indicated a purity of 99.8 percent; a test for chloride ion with silver nitrate was negative.

The benzoic acid was NBS Standard Sample 39g. Samples of the same material were used for both the calibration and the decomposition experiments.

The filter paper was Whatman No. 42. No attempt was made to dry the paper; instead it was stored at a constant humidity of 40 percent.

The oxygen was freed from traces of combustible materials and of carbon dioxide by passing it successively through (1) a tube packed with copper oxide and heated to 600 °C, and (2) an absorber containing Ascarite. No attempt was made to remove traces of nitrogen or water vapor.

## 3. Units of Energy and Molecular Weights

The unit of energy is the joule; for conversion to the conventional thermochemical calorie, one calorie is taken as 4.1840 joules.

All atomic weights were taken from the 1957 International Table of Atomic Weights [1].^1^

## 4. Apparatus and Procedure

The calorimeter was of the Dickinson isothermal- jacket type which has been described [2, 3]. The temperature of the calorimeter jacket was controlled within ±0.001 °C at about 27 °C by means of a thermostat. The calorimeter temperatures were measured by means of a platinum resistance thermometer and a Mueller-type bridge; timing of the experiments was made by reference to the NBS standard second signals.

The bomb was of the NBS twin-valve type, similar to that which has been described [3]; its volume was 375.5 ml. It was modified slightly for the KClO_4_ decomposition experiments in that two crucibles were used, one being mounted about 1 cm directly above the other. The KClO_4_ sample was weighed into the upper crucible. The benzoic acid pellet, approximately 0.6 g, was placed, in the lower crucible, on a strip of filter paper which extended out over the lip of the crucible. Ignition was accomplished by means of an electrically heated, iron-wire fuse which was placed in contact with the protruding end of the paper strip. One ml of water was placed in the bomb, which was then sealed, flashed with oxygen, and filled with oxygen to a pressure of 30 atm at 25 °C.

The details of the calorimetric procedure have been described [3, 4]. The heat evolved by the burning of the benzoic acid was sufficient to decompose the KClO_4_, leaving a fused mass of KCl in the crucible. In each case, a small portion of the KClO_4_ was blown out of the crucible during the decomposition; this made necessary a determination of the quantity of decomposition by analysis of the combustion products. The total mass of carbon dioxide was determined by collecting the dry gas in a weighed absorption tube containing Ascarite. The material remaining in the bomb was washed into a flask and the amount of chloride ion determined by titration with standard AgNO_3_, using fluorescein as the indicator [5].

The heat of combustion of the filter paper was determined in two separate experiments in which about 0.8 g of the paper was pressed into a wad, placed in the bomb, and burned in the usual manner. The mass of carbon dioxide produced by the combustion was determined as described above.

The calorimeter system was calibrated by a series of combustion experiments with benzoic acid, NBS Standard Sample 39g. The mass of benzoic acid was about 0.55 g in each instance; this value was chosen because it was sufficient for the KClO_4_-decomposition experiments and duplication, as nearly as possible, of the initial and final calorimeter temperatures was desirable.

## 5. Results and Calculations

The results of the calibration experiments with benzoic acid are given in table 1, where Δ*Rc* is the corrected rise in temperature of the calorimeter system (as measured on the particular thermometer and bridge [6]), *m_s_* is the mass of benzoic acid corrected to weight in vacuo, *q_i_* is the ignition energy from combustion of the iron-wire fuse, and *q_N_* is the energy evolved by the formation of nitric acid from traces of nitrogen in the bomb. The quantity *WC* is the Washburn correction [6, 7], applied here to convert the reactants and products from their thermodynamic standard states into the actual conditions of the bomb process; this correction may be defined by the relationship *WC*= Δ*E°−*Δ*E_B_.* The quantity Δ*e* is the deviation in the energy equivalent of the actual calorimeter system from that of the “standard” system; this value includes the heat capacity of the benzoic acid sample, a correction to the mean temperature of the calorimeter (26.5 °C) for the heat capacity of the calorimeter system, and a correction to 27.0 °C for ΔCv of the reaction of combustion.

The heat of combustion of benzoic acid for the standard bomb-process, −Δ*E_B_* (25 °C), was taken as 26433.8 j/g. This value was corrected to give −Δ*E°* (25 °C) = 26413.1 for the heat of combustion at constant volume, for the reactants and products in their thermodynamic standard states. A correction of 1.9 j/g made for ΔCv of the combustion reaction gave the following value for benzoic acid:

ΔE°(27°C)=−26411.2j/g

The energy equivalent of the “standard” calorimetric system for the temperature interval of 26 to 27 °C is obtained by:

$$E_{s}\;(27{}^{\circ}C)=\frac{26411.2\;m_{s}+q_{i}+q_{N}+WC}{\Delta Rc}-\Delta e.$$

The results of the experiments on the combustion of filter paper are given in table 2. The value for the standard combustion process at constant volume is given by:

$$-\Delta E{}^{\circ}\left(27{}^{\circ}C\right)=\left[\left(E_{s}+\Delta e\right)\Delta Rc-q_{i}-q_{N}+q_{t}-WC\right]/m_{fp}.$$

The quantity *q_t_* is a correction for the thermal coefficient of the combustion process to correct the experimental results to 27 °C. The value obtained for Δ*E°* (27 °C) was −16670 j/g.

The results of the experiments on the decomposition of KClO_4_ are given in table 3. The value obtained for the standard constant-volume process at 27 °C is given by the relationship:

$$-\Delta E{}^{\circ}\left(27{}^{\circ}C\right)=\frac{\left(E_{s}+\Delta e\right)\Delta Rc-q_{i}-q_{N}+q_{t}-q_{fp}-q_{ba}-WC}{\text{mole}\;{\text{KClO}}_{4}}$$

and may be represented by the equation:

$${\text{KClO}}_{4}\left(c\right)=\text{KCl}\left(c\right)+2O_{2}\left(g\right),$$ (1)

ΔE°(27°C)=−9.01±0.30kj/mole.

This result was corrected to constant pressure at 27 °C, with R = 8.3147 j/deg mole;

$$\begin{array}{c} \Delta H{}^{\circ}\left(27{}^{\circ}C\right)=\Delta E{}^{\circ}+\Delta \left(PV\right)=\Delta E{}^{\circ}+\Delta n\;RT\\ \;=-4.02\pm 0.30\;\text{kj}/\text{mole}. \end{array}$$

For eq (1) ΔC_p_ = −0.08 j/deg mole [8]; thus

$$\begin{array}{l} \Delta H{}^{\circ}\left(25{}^{\circ}C\right)=-4.02\pm 0.40\;\text{kj}/\text{mole}\\ \;=-0.96\pm 0.10\;\text{kcal}/\text{mole}. \end{array}$$

The uncertainty assigned to this value has been obtained by combining twice the standard deviations of the mean of the calibration and decomposition experiments with reasonable estimates for other errors.

The heat of formation of KCl(c) has been taken as −104.175 kcal/mole [8]. From this value and the heat of decomposition corresponding to eq (1) the following value has been obtained:

$${\text{KClO}}_{4}\left(c\right),\;\Delta \text{Hf}{}^{\circ}\left(25{}^{\circ}C\right)=-103.22\pm 0.10\;\text{kcal}/\text{mole}.$$

## 6. Discussion

Berthelot and Vieille [9] determined the difference between the heat of explosion of ammonium picrate and that of an ammonium picrate-potassium perchlorate mixture, and also the difference between the heat of explosion of potassium picrate and that of a potassium picrate-potassium perchlorate mixture. They obtained 7.5 kcal/mole for the decomposition of KClO_4_ according to eq (1).

Hofmann and Marin [10] burned a mixture of paraffin and potassium perchlorate and obtained ΔE(25 °C)= −1.73 kcal/mole for the constant- volume process corresponding to eq (1). Correction of their value for Δ(*PV*) gives ΔH (25 °C) = −0.55 kcal/mole.

Rossini, Wagman, Evans, Levine, and Jaffe [8] used the data of Hofmann and Marin because they were the only data available which were reasonably compatible with the data on potassium chlorate and on perchloric acid.

## Figures and Tables

**Table 1:** Results of the calibration experiments with benzoic acid

Experiment No.	Δ*Rc*	*m_s_*	*q_i_*	*q_N_*	*WC*	Δ*e*	*E_s_*

	*ohm*	*g*	*j*	*j*	*j*	*j/ohm*	*j*/*ohm*
1	0.114219	0.61115	34.6	0.9	11.8	3.9	141728.3
2	.103961	.55620	34.6	1.1	10.9	7.2	141743.2
3	.103602	.55427	34.0	1.2	10.8	7.2	141736.5
4	.101401	.54238	35.7	0.4	10.3	7.1	141720.4
5	.101426	.54252	35.6	1.0	10.5	7.1	141728.8

Mean							141731.4
Standard deviation of the mean							±3.9

**Table 2:** Results of the experiments on the combustion of filter paper

Experiment No.	Δ*Rc*	Δ*e*	*q_i_*	*q_N_*	*q_t_*	*WC*	*m_fp_*	*−*ΔE°(27 °C)

	*ohm*	*j*/*ohm*	*j*	*j*	*j*	*j*	*g*	*j*/*g*
1	0.098558	16.6	31.4	0.7	−0.2	10.5	0.83479	16684.0
2	.091101	17.1	32.2	1.4	0.0	9.6	.77271	16656.0

Mean								16670.0

**Table 3:** Results of the experiments on the decomposition of KClO_4_

Experiment No.	Δ*Rc*	Δ*e*	*q_i_*	*q_N_*	*q_t_*	*q_fp_*	*q_ba_*	*WC*	KClO_4_	*−*ΔE° (27 °C)

	*ohm*	*j*/*ohm*	*j*	*j*	*j*	*j*	*j*	*j*	*mole*	*kj*/*mole*
1	0.114725	45.6	32.4	0.9	0.1	216.4	15873.9	21.9	0.01336	8.980
2	.104967	45.0	34.6	.9	.1	226.9	14524.5	17.5	.00944	8.214
3	.108221	37.3	32.5	.9	.1	287.7	14881.6	25.5	.01301	8.782
4	.113302	42.5	32.6	.9	.1	269.6	15620.3	21.8	.01334	8.858
5	.114737	38.0	33.3	.9	.1	223.9	15848.5	23.3	.01496	9.117
6	.114943	37.5	32.0	.9	.1	227.5	15877.3	22.9	.01436	9.390
7	.114876	37.5	35.0	.9	.1	231.2	15866.5	22.8	.01422	9.110
8	.114358	37.5	33.5	.9	.1	184.2	15852.8	21.2	.01244	9.639

Mean										9.011
Standard deviation of the mean										±0.151

## Footnotes

1 Figures in brackets indicate the literature references at the end of this paper.
